# Preparation, Characterization and Dielectric Properties of Alginate-Based Composite Films Containing Lithium Silver Oxide Nanoparticles

**DOI:** 10.3389/fchem.2021.777079

**Published:** 2022-01-18

**Authors:** Padma Giriyappa Thimmaiah, Venkata Ramana Mudinepalli, Subba Rao Thota, Sreekanth Reddy Obireddy, Wing-Fu Lai

**Affiliations:** ^1^ Materials Research Laboratory, Department of Physics, Sri Krishnadevaraya University, Anantapur, India; ^2^ Department of Physics, National Taiwan Normal University, Taipei, Taiwan; ^3^ Department of Chemistry, Sri Krishnadevaraya University, Anantapur, India; ^4^ Department of Applied Biology and Chemical Technology, Hong Kong Polytechnic University, Hong Kong Special Administrative Region, Hong Kong, China; ^5^ Ciechanover Institute of Precision and Regenerative Medicine, The Chinese University of Hong Kong (Shenzhen), Shenzhen, China; ^6^ Department of Urology, Zhejiang Provincial People’s Hospital, Affiliated People’s Hospital, Hangzhou Medical College, Zhejiang, China

**Keywords:** composite films, sodium alginate, lithium silver oxide, dielectric properties, energy storage

## Abstract

Polymer composites have found applications in diverse areas, ranging from the manufacturing of portable electronic devices to the fabrication of bioactive agent carriers. This article reports the preparation of composite films consisting of sodium alginate (SA) and lithium silver oxide (LAO) nanoparticles. The films are generated by solution casting; whereas the nanoparticles are fabricated by using the hydrothermal method. The effects of the nanoparticles on the morphological, thermal, and dielectric properties of the films are examined by using Fourier transform infrared (FTIR) spectroscopy, X-ray diffraction (XRD) analysis, differential scanning calorimetry (DSC), thermogravimetric analysis (TGA), and scanning electron microscopy (SEM). Electrical measurements are also performed to determine the dielectric constant (ε′), dielectric loss (ε″), AC conductivity (σ_ac_), electrical moduli (M′ and M″), and impedance (Z^'^ and Z″). The composite films are shown to be crystalline in nature, with nanoparticles having a diameter of 30–45 nm effectively disseminated in the polymer matrix. They also display good dielectric properties. Our results suggest that the films warrant further exploration for possible use in microelectronic applications.

## Introduction

Polymer composites have found applications in a large variety of areas, ranging from the generation of portable electronic devices and high-speed integrated circuits (Deshmukh et al., 2017; Zhang et al., 2002) to the development of drug delivery systems (Lai et al., 2018; Lopez-Lugo et al., 2021; Rajesh et al., 2021). This is partially attributed to good processability and high structural flexibility of polymer composites (Chen et al., 2015; Choudhary and Sengwa, 2017; Dang et al., 2012; Xu et al., 2017). Since the turn of the last century, many researchers have investigated the dielectric properties of various polymer composites (Kavitha et al., 2017; Maharramov et al., 2018), and have found that such properties are affected by the chemical composition, crystallinity, and morphological features of the composites (Sunilkumar et al., 2014). Proper optimization of the properties of the polymer and the inorganic fillers is, therefore, required for the attainment of polymer composites with maximal performance (Balazs et al., 2006; Deshmukh et al., 2017).

**GRAPHICAL ABSTRACT:**
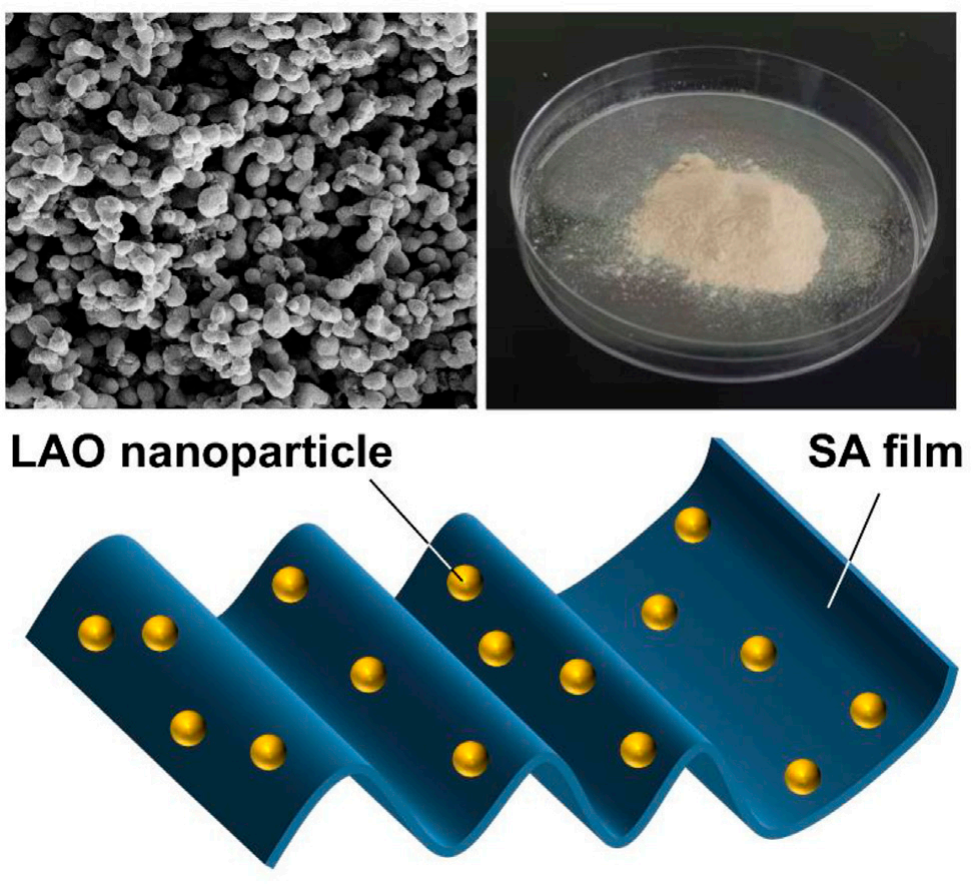


As far as the selection of inorganic fillers is concerned, metal oxides and metal nanoparticles (NPs) (including silver NPs, copper NPs, copper oxide, aluminium oxide, lithium aluminium oxide, iron oxide, barium titanium oxide, and titanium oxide) have been brought forward because they enable the generation of polymer composites with high electrical conductivity (Afzal et al., 2008; Chen et al., 2015; Choudhary and Sengwa, 2017; Dang et al., 2012; Deshmukh et al., 2017; Huang et al., 2009; Li et al., 2009; Li, et al., 2017; Luo et al., 2014; Nelson et al., 2002; Xu et al., 2017; Yang et al., 2015; Zhang et al., 2016; Zhang et al., 2002). The electrical properties of the composites are determined not only by the size and morphology of the NPs (Masoud et al., 2013) but also by the interactions between NPs and the polymer matrix (Deshmukh et al., 2017; Yang et al., 2015). Because of the latter, various strategies (including surface modification (Riggs et al., 2015; Zhang et al., 2016) and ultrasonication of the NPs (Xu et al., 2017) have been adopted to enhance dispersion of the NPs in the polymer matrix.

Apart from the inorganic fillers, the polymer moiety plays an important role in determining the ultimate properties of the composites generated. One of the most extensively studied polymers is sodium alginate (SA), which is a naturally occurring anionic polysaccharide derived from brown seaweeds (Lai et al., 2016; Reddy et al., 2020; Sreekanth Reddy et al., 2021). Besides its use in composite fabrication (Bibi et al., 2020; Kloster et al., 2020; Leonardi et al., 2021), SA has been widely exploited for biomedical and electrical applications due to its low cost, high biodegradability and negligible toxicity (Lai et al., 2020a; Lai et al., 2020b; Praveena et al., 2014; Rachocki et al., 2011). By taking advantage of these favourable properties of SA, in this study we have fabricated a series of biodegradable composite films. The films are made by using solution casting, and are incorporated with lithium silver oxide (LAO) NPs. Because of the NPs used in this study are synthesized by using the hydrothermal process, not only can the NPs be attained at low cost but more precise control over the particle size can be achieved (Ji et al., 2019; Ji et al., 2020). To the best of our knowledge, this is one of the first studies reporting the hydrothermal synthesis of LAO NPs and the use of LAO NPs for the preparation of SA-based composite films with good dielectric properties.

## Experimental

### Synthesis of LAO NPs

LAO NPs were generated from Li(NO_3_) and Ag(NO_3_) by using the low-temperature hydrothermal technique. 0.527 g of Li(NO_3_) and 1.2986 g of Ag(NO_3_) were dissolved in 10 ml of distilled water under constant magnetic stirring for 3 h. The pH of the solution was adjusted to 12 by adding an NaOH solution dropwise. After that, the solution was placed in a Teflon-lined steel autoclave and heated in an oven at 140°C for 10 h. After cooling to room temperature, the autoclave was opened under normal atmosphere. The solution was centrifuged at 10,000 rpm for 10 min. The residue obtained was washed with acetone and distilled water several times, and dried at 60°C for 2 h to obtain LAO NPs.

### Synthesis of SA-LAO Composite Films

Five gram of SA was dissolved in 90 ml of distilled water and stirred at ambient conditions for 48 h. 0.1 g of LAO NPs was dispersed in 10 ml of distilled water and added into the SA solution. Upon sonication for 30 min, the solution was stirred for additional 6 h. Afterwards, the solution was poured into a petri dish and air-dried at ambient conditions. The film obtained was designed as SA2 and stored in a desiccator until required for analysis. The same procedure was adopted to generate films containing different amounts (4, 8, 16 wt% of SA) of LAO NPs. The generated films were designated as SA4, SA8, and SA16, respectively.

### Structural and Physical Characterization

X-ray diffraction (XRD) analysis of the films was carried out using an X-ray diffractometer (Bruker AXS D8; Rigaku Corporation, Japan, Tokyo) outfitted with Cu-Kα radiation (*λ* = 1.5406 A). The accelerating voltage and current were 40 kV and 40 mA, respectively. These films were scanned in the 2θ range of 10–60° at a scanning speed of 2°/min. Thermogravimetric analysis (TGA) and differential scanning calorimetry (DSC) were performed by using a simultaneous thermal analyser (STA) (Q600; TA Instruments, New Castle, Delaware, United Kingdom) to study the thermal properties of the composite films in the temperature range of 30–600°C at a heating rate of 10°/min under nitrogen atmosphere. Fourier transform infrared (FTIR) spectra of the samples embedded in KBr pellets were recorded using a FTIR spectrophotometer (Impact 410; Nicolet Analytical Instruments, Milwaukee, WI, United States). The morphological features and elemental composition of the samples, upon gold sputtering, were examined by scanning electron microscopy (SEM) equipped with energy-dispersive spectroscopy (EDS) (ULTRA 55; Carl Zeiss, Oberkochen, Germany). The electrical properties of the films were analysed by using a computer controlled impedance analyser (LCR HiTester3532-50; Hioki Corporation, Nagano, Japan) over the frequency range from 100 Hz to 5 MHz and over the temperature from 40°C to 100°C.

## Results and DISCUSSIONS

### Structural Properties of the Films

**FIGURE 1:**
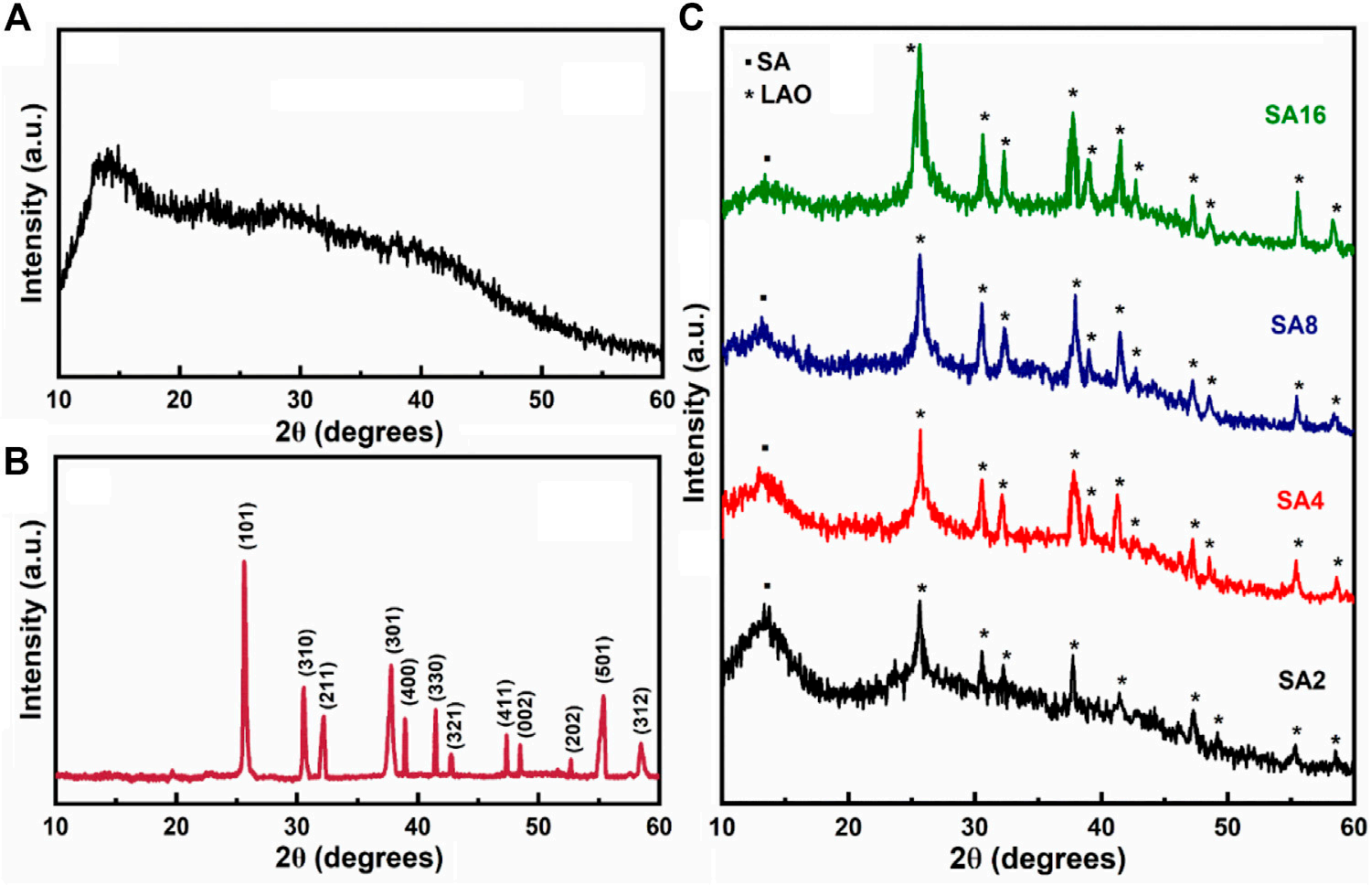
XRD diffractograms of **(A)** the SA film, **(B)** LAO NPs, and **(C)** the composite films.

The XRD diffractogram of the SA film shows a broad peak at 2θ = 13.32° (Figure 1A), indicating the presence of an amorphous structure. The interplanar spacing is estimated to be 6.63 Å by using the equation *nλ* = 2*d* sin*θ* (where *λ* is the wavelength, *d* is the interplanar spacing, and *θ* is the diffraction angle of the corresponding peak) (Ionita et al., 2013; Rhim and Kim, 2000). Figure 1B shows the diffraction pattern of LAO NPs. The sharp reflection peak at 2θ = 25.61°, along with other diffraction peaks matched with JCPDS (36–1070), reveal the pure tetragonal structure of the NPs. The lattice parameters for the tetragonal structure are calculated using the following equation (Eq. 1):

$$\frac{1}{d^{2}}=\frac{\left(h^{2}+k^{2}\right)}{a^{2}}+\frac{l^{2}}{c^{2}}$$ (1)

where *a* and *c* are lattice parameters of the corresponding lattice, (*hkl*) are miller indices of the lattice planes. The lattice parameters (*a* = 9.253 and *c* = 3.753) are determined based on the *d*-spacing and miller indices of the maximum intensity peak (101) and the subsequent peak (310). These values are consistent with the values (a = 9.248, c = 3.750) reported in the JCPDS card no. 36–1070. The crystallite size (D) of the LAO NPs can be deduced using the Scherer’s equation (Eq. 2) (Bouazizi et al., 2014; Siddiqui et al., 2018):

D=kλ/βcosθ (2)

where *k* = 0.9 is a constant and *β* is the full width at half-maximum corresponding to the Braggs angle of diffraction *θ*. Based on the most intense diffraction peak (101), the average crystal size is calculated to be around 31 nm. The XRD diffractograms of the composite films containing different amounts of NPs are shown in Figure 1C. The diffraction peaks from LAO NPs are found in the diffraction patterns of all of the film samples. This implies that the structure of the NPs remains unchanged, and that the films are crystalline in nature (Afzal et al., 2008; Ionita et al., 2013; Sengwa and Choudhary, 2016). When the amount of LAO NPs in a film increases, the peak at 2θ = 13.32° shows a decrease in intensity but an increase in broadness.

**FIGURE 2:**
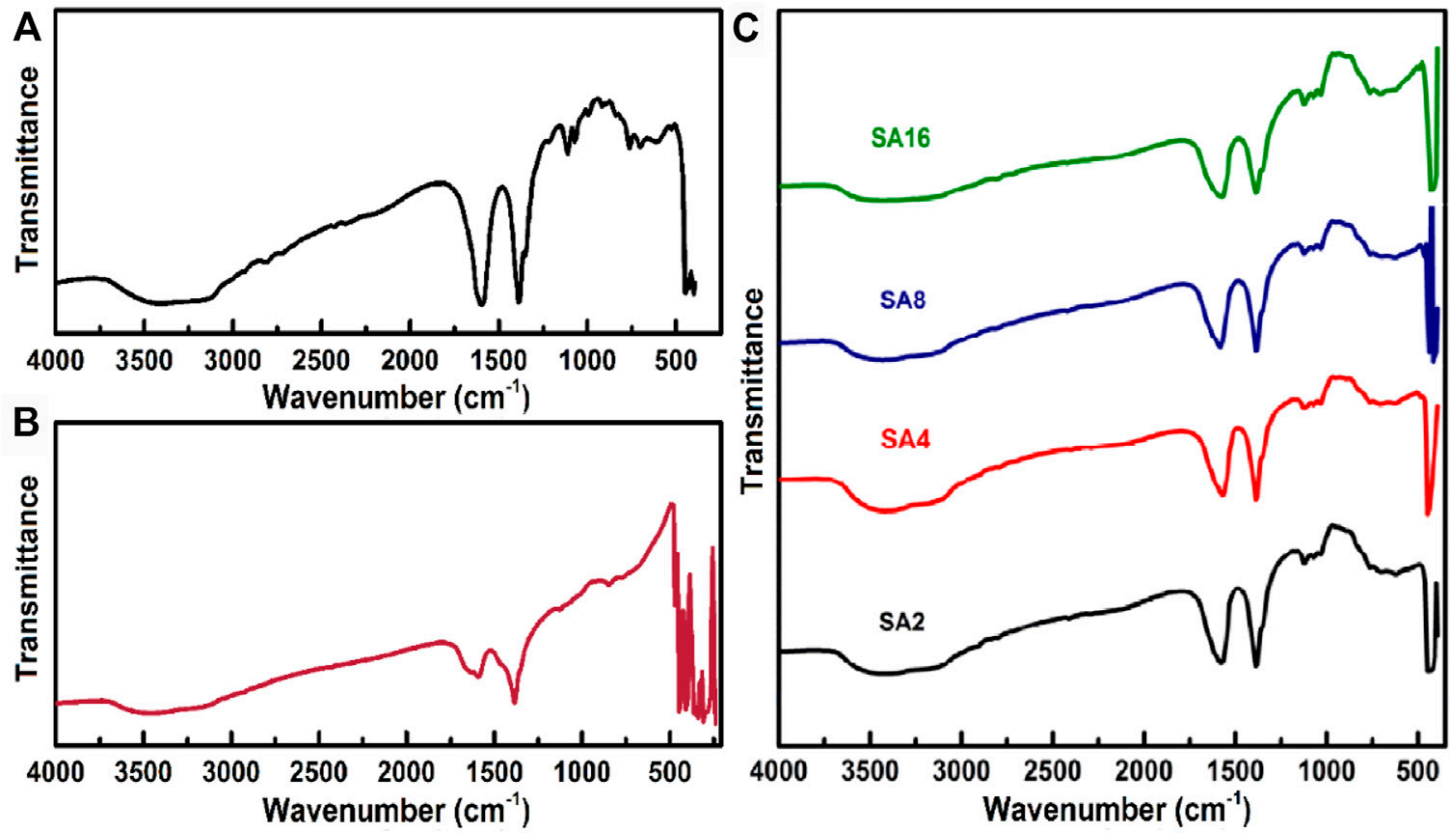
FTIR spectra of **(A)** the SA film, **(B)** LAO NPs, and **(C)** the composite films.

To examine the specific molecular interactions between the SA matrix and the NPs, FTIR spectroscopy is adopted (Figure 2). The spectrum of the SA film displays a broad peak at 3394 cm^−1^. This peak is assigned to the stretching vibrations of the OH group (Ionita et al., 2013; Sheela et al., 2016). The peak at 2812 cm^−1^ is due to the symmetric stretching vibrations of the C-H group; whereas peaks at 1603 and 1388 cm^−1^ are attributed to the asymmetric stretching vibrations of the carboxyl group (Liu et al., 2014; Shameem et al., 2018). The peaks at 1111 and 1064 cm^−1^ belong to the stretching vibrations of the C-C group (Li et al., 2011; Russo et al., 2010; Sartori et al., 1997). The peaks at 985, 908, and 838 cm^−1^ are due to the existence of guluronic and mannuronic acid units. In the FTIR spectrum of LAO NPs, three prominent peaks are found at 3452, 1603, and 1382 cm^−1^. These peaks are assigned to the stretching, bending, and deformation vibrations of the OH group, respectively. Their existence is due to the moisture absorbed in the NPs. The peaks at 472, 442, 407, and 346 cm^−1^ are assigned to the metal-oxygen bond in LAO. These peaks are found in the FTIR spectra of the composite films. In addition, in comparison with those in the spectrum of the SA films, the peaks corresponding to the hydroxyl and carboxyl groups slightly shift towards the lower wavelength side in the spectra of the composite films (Table 1). The shift of the peak corresponding to the hydroxyl group is caused by hydrogen bonding interactions between SA and LAO (Srivastava et al., 2012); whereas that of the peak corresponding to the carboxyl group is due to the symmetrical carboxylate bonding of SA to the LAO NP surface (Unal et al., 2010; Srivastava et al., 2012). The incorporation of LAO into the SA film also leads to a decrease in the intensity of stretching vibrations of the OH group, suggesting that the NPs are dispersed in the SA matrix (Elizabeth et al., 2004; Suhas et al., 2013).

**TABLE 1:** The position of the peaks corresponding to the hydroxyl and carboxyl groups in the FTIR spectra of the film samples.

Film	Wavenumber (cm^−1^)	
	Hydroxyl group	Carboxyl group
SA	3409	1605
SA2	3389	1596
SA4	3384	1589
SA8	3379	1581
SA16	3371	1573

### Thermal Properties of the Films

**FIGURE 3:**
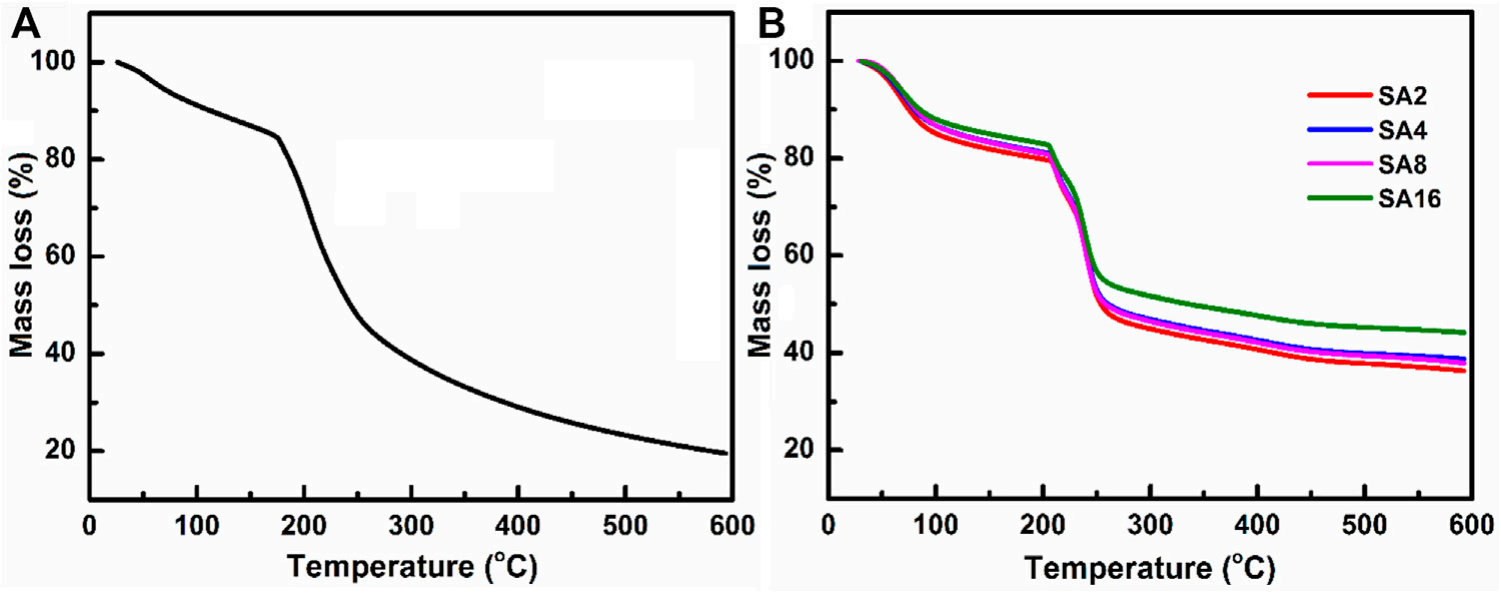
TGA curves of **(A)** the SA film and **(B)** the composite films.

TGA is adopted in this study to determine the thermal stability and weight loss behaviour of the film samples (Figure 3). In the TGA curves of the films, a multi-step thermal degradation process is observed. The temperature at which weight loss occurs abruptly is considered to be the degradation temperature (Sheela et al., 2016). The SA film exhibits a weight loss step (∼15%) between 30 and 100°C due to the dehydration of the sample (Bekin et al., 2014; Shameem et al., 2018). Another weight loss step (∼35%) is observed between 175 and 245°C. This is caused by the decomposition of the SA backbone (Xiao et al., 2002; Tripathi and Mishra, 2012). Upon the incorporation of the NPs, the degradation temperature and hence the thermal stability of the SA film increase. The decomposition temperature of the composite films lies in the temperature range from 204–208°C. In addition, no significant weight drop is observed above 450°C. The percentage of weight loss experienced by the composite films is 30% lower than that of the SA film, with the residual mass of the composite films reaching 40–50% at 600°C.

**FIGURE 4:**
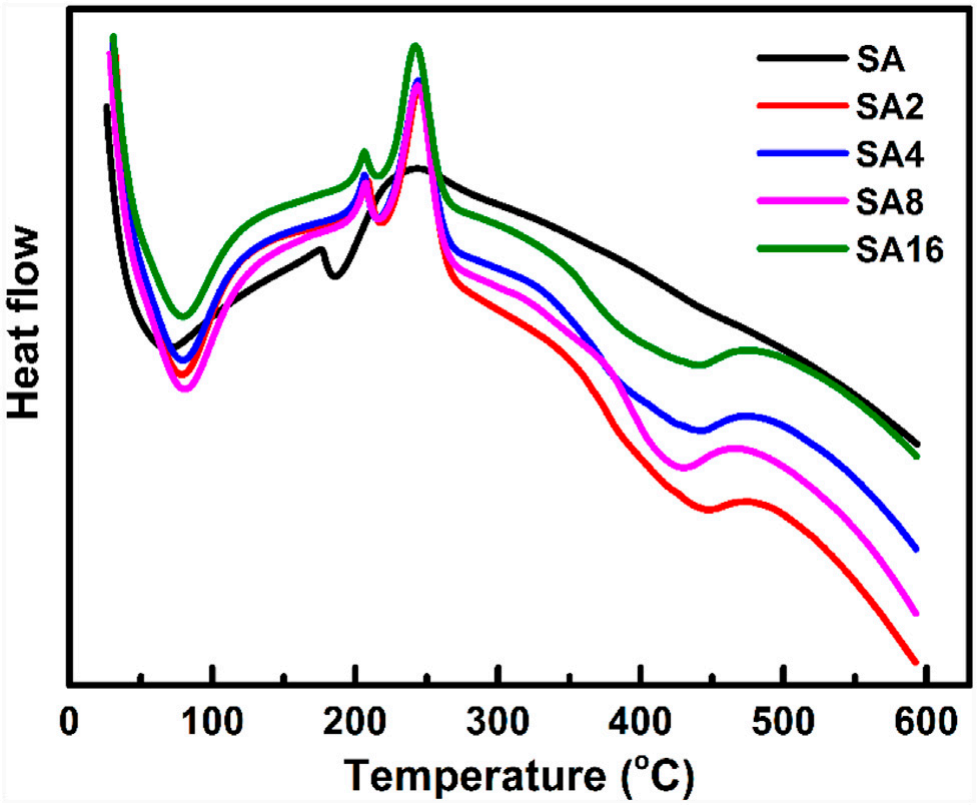
DSC thermograms of different film samples.

To investigate the melting process of the film samples, DSC is used (Figure 4). Two endothermic peaks are found in the curve of the SA film. The endothermic peak at 60°C is caused by the evaporation of the absorbed moisture in the film (Smitha et al., 2005; Agrawal et al., 2019). The endothermic peak at 180°C corresponds to the melting temperature (T_m_) of the SA film (Agrawal et al., 2019). In the curves of the composite films, the T_m_ value increases with the LAO content of the film. Comparing with the T_m_ of the SA film, that of the composite films is much higher. This, along with the results of TGA as discussed above, reveals that incorporation of LAO NPs into the SA matrix enhances the thermal stability of the SA film, with the enhancing effect not only relying on the NP content but also on the interactions between the NPs and the polymer matrix (Raghavendra et al., 2013; Deshmukh et al., 2017).

### Morphological and Compositional Features of the Films

**FIGURE 5:**
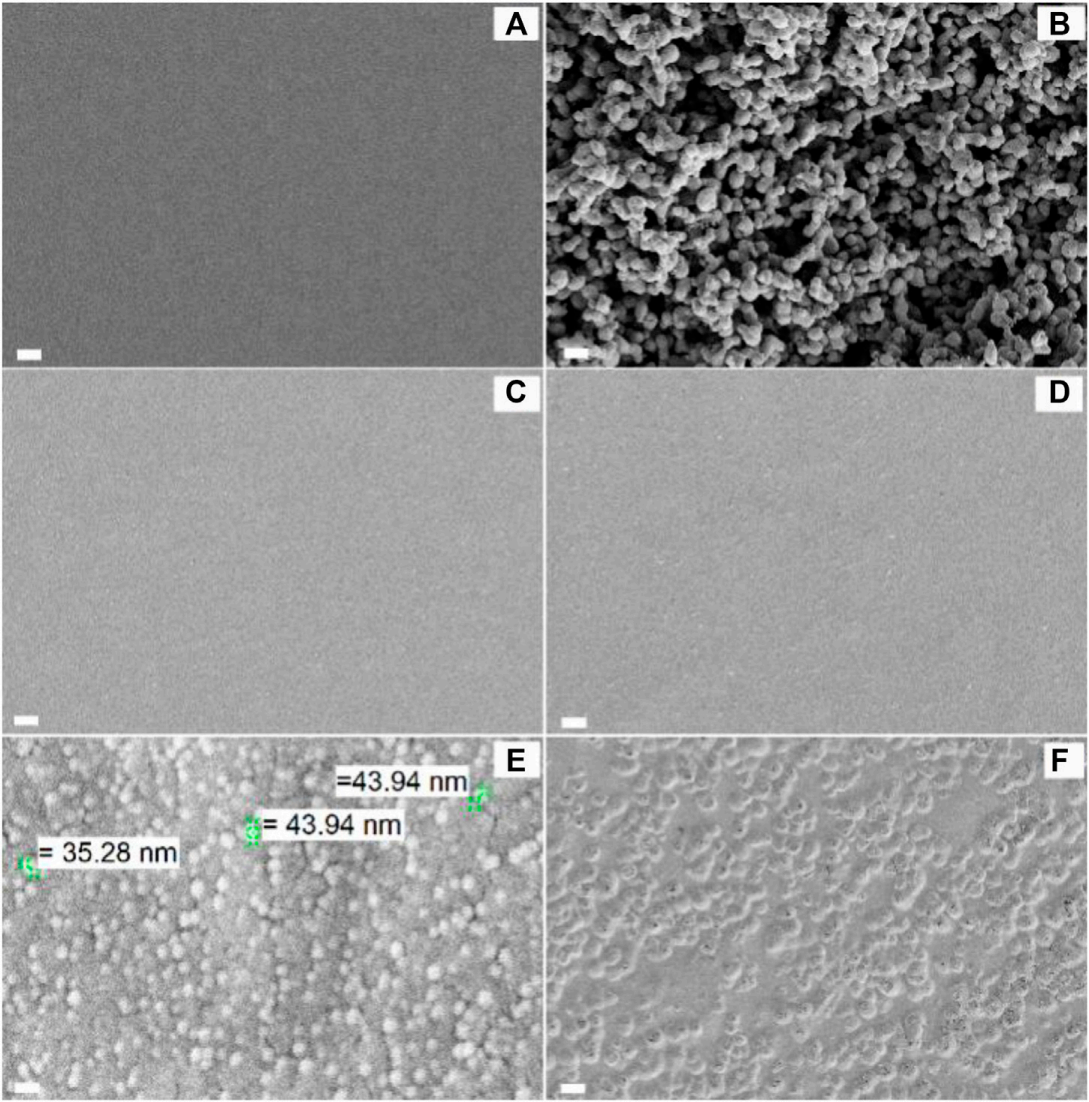
SEM images of **(A)** the SA film, **(B)** LAO NPs, and different composite films: **(C)** SA2, **(D)** SA4, **(E)** SA8, and **(F)** SA16. The scale bar in **(A)**, **(C)**, **(D)** and **(F)** is 10 µm. The scale bar in **(B)** and **(E)** is 50 nm.

**FIGURE 6:**
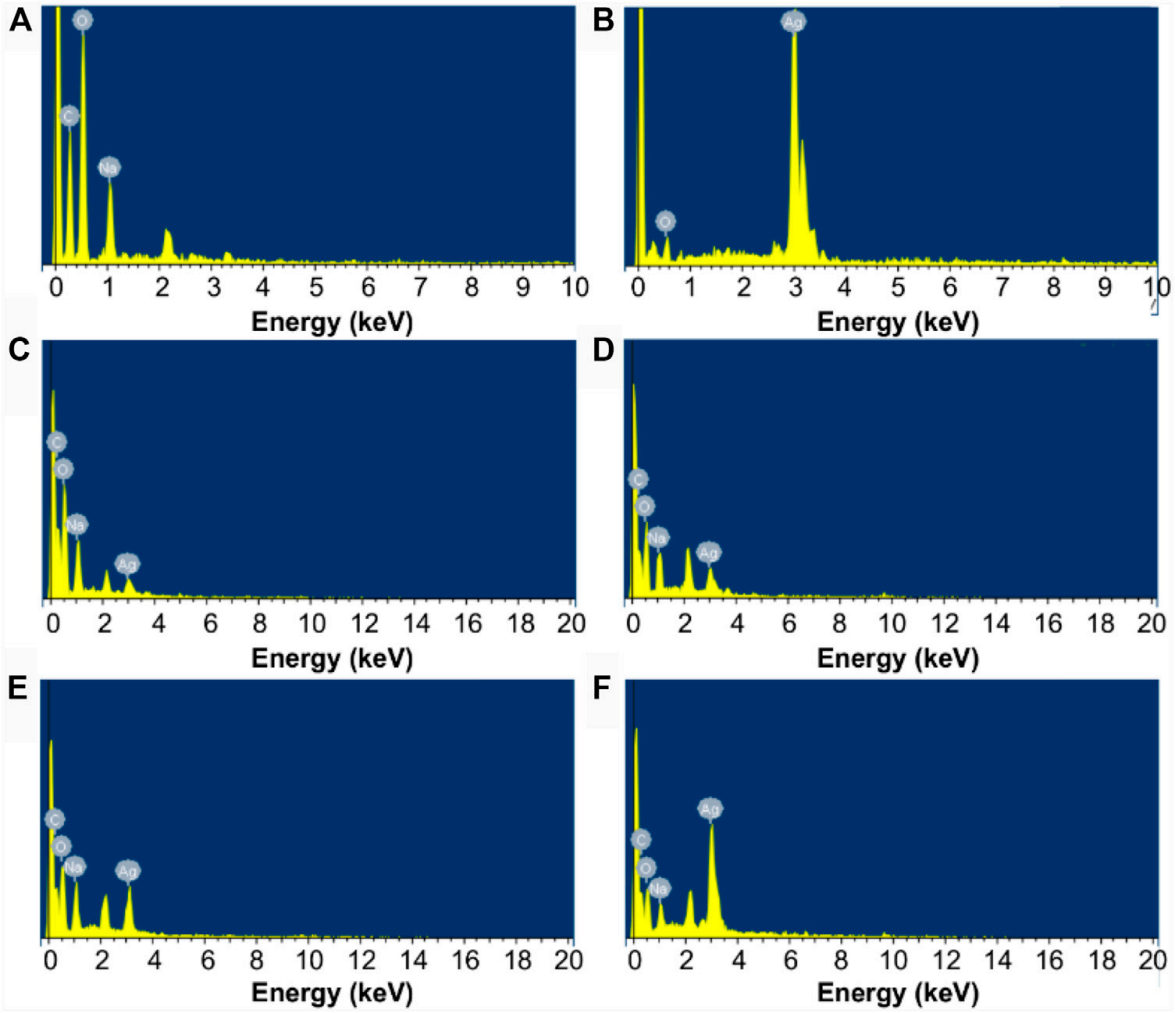
EDS spectra of **(A)** the SA film, **(B)** LAO NPs, and different composite films: **(C)** SA2, **(D)** SA4, **(E)** SA8, and **(F)** SA16.

The morphological features of the LAO NPs and the film samples are examined by using SEM (Figure 5). The SEM micrograph of the SA film shows that the surface of the film is homogeneous (Figure 5A). This is consistent with the observation previously reported by Suhas et al. (2014) and Ionita et al. (2013). In the SEM micrograph of LAO NPs, the sample shows a homogeneous structure with uniform size distribution, with the average diameter of the NPs being 30–45 nm (Figure 5B). Upon the incorporation of the NPs into the SA matrix, an increase in the surface roughness of the SA film is observed, though no phase separation is noted (Figures 5C–F). The degree of surface roughness is positively related to the concentration of LAO NPs in the film sample. The chemical composition of the films is analysed by using EDS (Figures 6A–F). Major elements corresponding to SA and LAO NPs are detected in all of the composite films, with the intensity of the signal corresponding to silver being positively related to the NP content. Based on the results presented above, LAO NPs are dispersed homogeneously in the SA matrix. Strong interfacial interactions are anticipated to play a role in enhancing the dielectric performance of our SA-LAO composite films.

### Changes of the Dielectric Constant and Dielectric Loss with Frequency and Temperature

Dielectric properties play an important role in determining energy storage capacity and molecular mobility in composite films (Bekin et al., 2014; Abdel-Baset et al., 2016; El-Ghamaz et al., 2016). The dielectric constant (ε*) is a complex value, with *ε′* denoting the real part and *ε"* denoting the imaginary part (Eq. 3):

$$\varepsilon^{*}=\varepsilon^{\prime }-i\varepsilon^{\prime \prime }$$ (3)

The real part of the dielectric constant refers to energy storage in a material, and can be determined using the following equation (Eq. 4) (Chandrakala et al., 2013; El-Ghamaz et al., 2016):

$$\varepsilon^{\prime }=C_{p}d/\varepsilon_{0}A$$ (4)

where *C*_*p*_ is the parallel capacitance, *d* is the thickness of the circular film, *A* is the cross-sectional area of the film, *ε*_*o*_ is the permittivity of free space. The dielectric loss *ε"* refers to energy loss in a material and can be calculated using the following equation (Eq. 5) (Chandrakala et al., 2013; El-Ghamaz et al., 2016).

$$\varepsilon^{\prime \prime }=\varepsilon^{\prime }\tan\delta$$ (5)

where tan*δ* is the tangent loss. The dielectric behaviour of a composite film is the result of different polarization mechanisms (including dipolar polarization, ionic polarization, and interfacial polarization) adopted by the films under an alternating electric field (Choudhary and Sengwa, 2017; Deshmukh et al., 2017).

**FIGURE 7:**
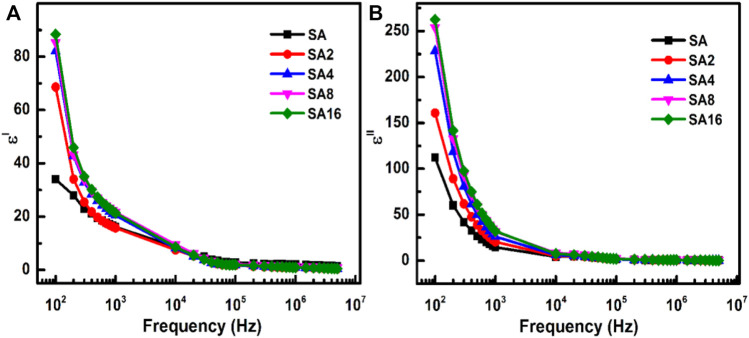
The frequency dependence of the **(A)** dielectric constant and **(B)** dielectric loss of different films at room temperature.

The dielectric properties of our alginate-based composite films are studied as a function of frequency, ranging from 100 Hz—5 MHz (Figure 7). The ε′ and ε″ values of the films are found to decrease with an increase in frequency, with a constant value being attained when the frequency reaches 100 kHz or above. This observation can be explained by Koop’s theory (Koops, 1951), which states that a polycrystalline material consists of grains and grain boundaries, with the former being less resistive than the latter. When a material is subjected to an alternating electric field, charge carriers tend to accumulate at the grain boundary interfaces, resulting in the formation of dipoles. The reinforcement of charge carriers results in Maxwell Wagner interfacial or space charge polarization (Yager, 1936; Koops, 1951; Chandrakala et al., 2013; El-Ghamaz et al., 2016). For this, when the frequency is low, more charges in the composite film are trapped at the interfaces because of the high resistive nature of the grain boundaries. This leads to space charge polarization, causing the ε′ and ε″ values to be high. When the frequency increases, charge carriers less likely accumulate at the interfaces. This leads to a decrease in the ε′ and ε″ values. Such a mechanism is termed anomalous dielectric dispersion (Wahab et al., 1997; Reddy et al., 2010; Praveena et al., 2014; Abbas et al., 2015).

Compared to the composite films, the SA film possesses a lower ε′ value (Figure 7A). Furthermore, in composite films, the ε′ value increases with the concentration of LAO NPs present. This is attributed to interfacial polarization, in which more charge carriers are trapped at the interfaces because more free charges are available at the interfaces upon doping. Similar to the case of ε′, the ε″ value decreases as the frequency increases. This is because when the frequency is low, electric dipoles have more time to orient themselves in the field direction, leading to a high ε″ value. Because of this, incorporation of LAO NPs into the film increase the ε″ value when the frequency is low (100 Hz) but suppresses the ε″ value when the frequency is high (5 MHz). This phenomenon is desired for embedded passive applications (Sui et al., 2009; Deshmukh et al., 2017).

**FIGURE 8:**
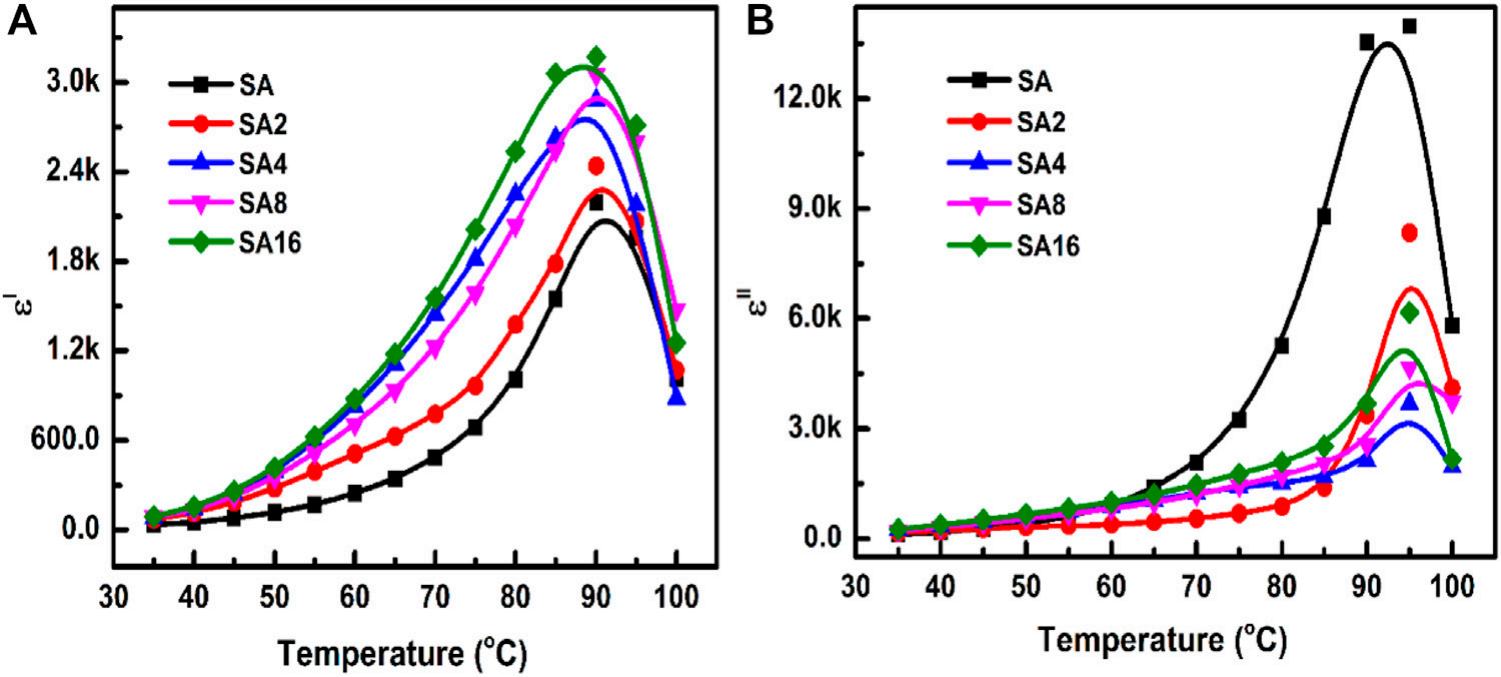
The temperature dependence of the **(A)** dielectric constant and **(B)** dielectric loss of different films at 100 Hz.

The temperature dependence of the *ε′* and *ε"* values of different films at 100 Hz is evaluated from room temperature to 100°C (Figure 8). As the temperature increases, ε′ increases. This is because an increase in temperature not only makes electric dipoles more effective in orientating themselves in the field direction (Schildknecht and Finch, 1974; Hanafy, 2008; Masoud et al., 2013; Abdel-Baset et al., 2016) but can also result in thermal activation of the charge carriers to lead to an increase in polarization (Sengwa and Choudhary, 2016). In addition, as the specific volume of the polymer increases, a small amount of the crystalline phase gets dissolved and changes into an amorphous phase, resulting in a higher *ε′* value (Rao et al., 2000; Awadhia et al., 2006). Compared to the SA film, our composite films have a higher *ε′* value. This observation is consistent with previous studies in which an increase in *ε′* has been noted upon the incorporation of metal oxides into a polymer (Prabu and Selvasekarapandian, 2012; Abdel-Baset et al., 2016; Sengwa and Choudhary, 2016; Choudhary and Sengwa, 2017). The high *ε′* value of our composite films is contributed by the even dispersion of LAO NPs in the SA matrix and by the high εʹ value of LAO NPs. Similar to the case of *ε′*, the value of dielectric loss increases with temperature in all of the tested films. A peak corresponding to phase transition is found at around 90°C. This phase transition is indeed the glass transition experienced by the films.

### Changes of AC Conductivity with Frequency and Temperature

AC conductivity (*σ*_*ac*_) of a film is calculated by using Eq. 6, where *ω* is the angular frequency. Meanwhile, it is affected by temperature. This effect is governed by the Arrhenius relation (Abdel-Baset et al., 2016; Sengwa and Choudhary, 2016), which can be determined by using Eq. 7, where *σ*_*o*_ is the pre-exponential factor, *E*_*a*_ is the activation energy, *k* is the Boltzmann constant, *T* is the absolute temperature.

$$\sigma_{ac}=\varepsilon_{0}\varepsilon^{\prime \prime }\omega$$ (6)

$$\sigma_{ac}=\sigma_{0}^{\frac{-Ea}{kT}}$$ (7)

**FIGURE 9:**
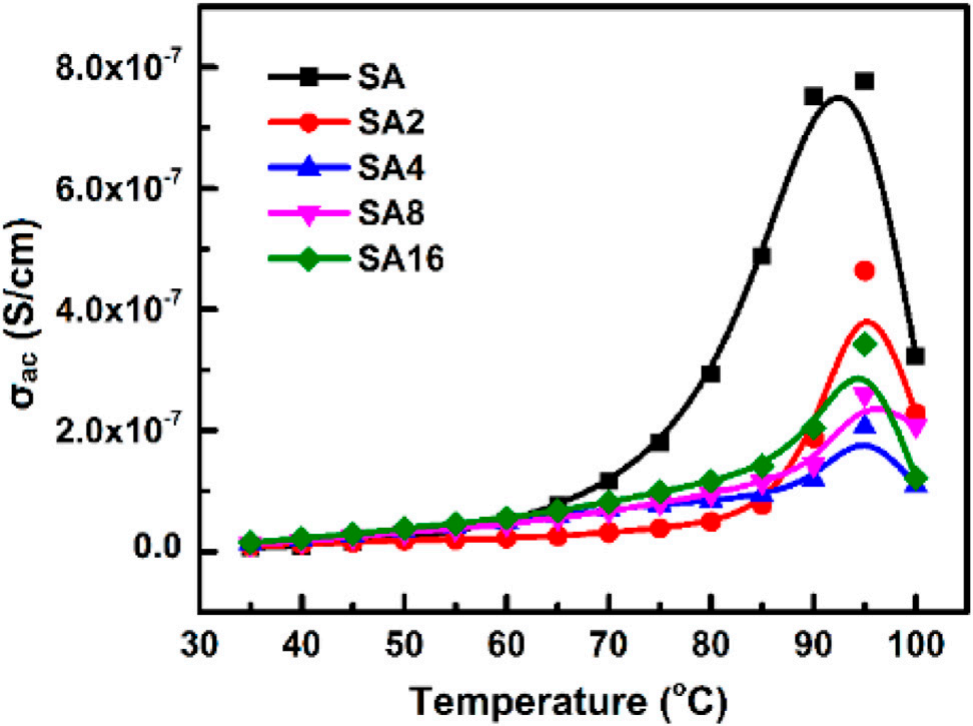
The temperature dependence of AC conductivity of different films at 100 Hz.

**FIGURE 10:**
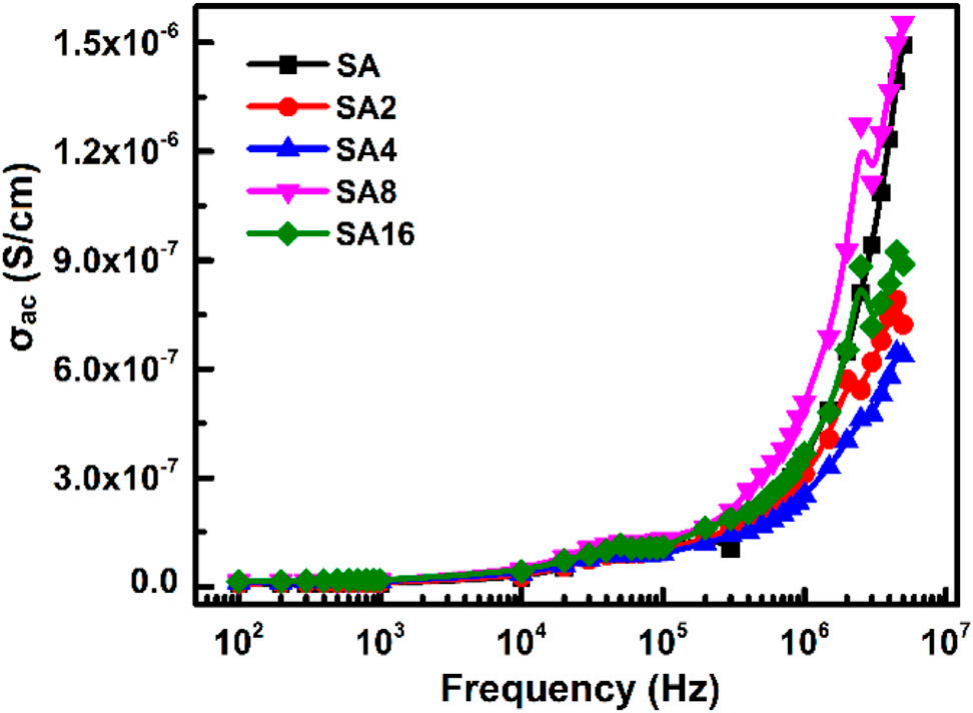
The frequency dependence of AC conductivity of different films at room temperature.

The temperature dependence of *σ*_ac_ of our films is shown in Figure 9. Based on the result, *σ*_ac_ increases with temperature in all films tested. This is explained by the fact that an increase in temperature enhances the movement of inter-chain and intra-chain charge carriers (Baskaran et al., 2006; El-Ghamaz et al., 2016; Sheela et al., 2016; Sherman et al., 1983), resulting in an increase in AC conductivity. Furthermore, *σ*_ac_ of the SA film decreases upon the incorporation of LAO NPs. This is because the film becomes more crystalline in nature when LAO is added. Polymer chains have lower flexibility in the crystalline state. This reduces AC conductivity (Correa et al., 2017; Mathew et al., 2015; Ratner & Shriver, 1988). The sudden drop in σ_ac_ at 100°C is possibly due to experimental error, which is resulted from the fact that 100°C is the temperature limit of the measurement system. Besides temperature, the frequency of the electric field is a factor affecting *σ*_ac_ of our films (Figure 10). The frequency-dependent increase in *σ*_ac_ is resulted from the hopping of charge carriers and from the fact that electric dipoles oscillate with a higher velocity as the frequency increases. A similar observation has been made by other studies on polymer composites including the polyvinyl chloride/silica composite film (Abdel-Baset et al., 2016) and the poly(vinylidene chloride-co-acrylonitrile)/poly(methyl methacrylate) blend membrane (Mathew et al., 2015).

**FIGURE 11:**
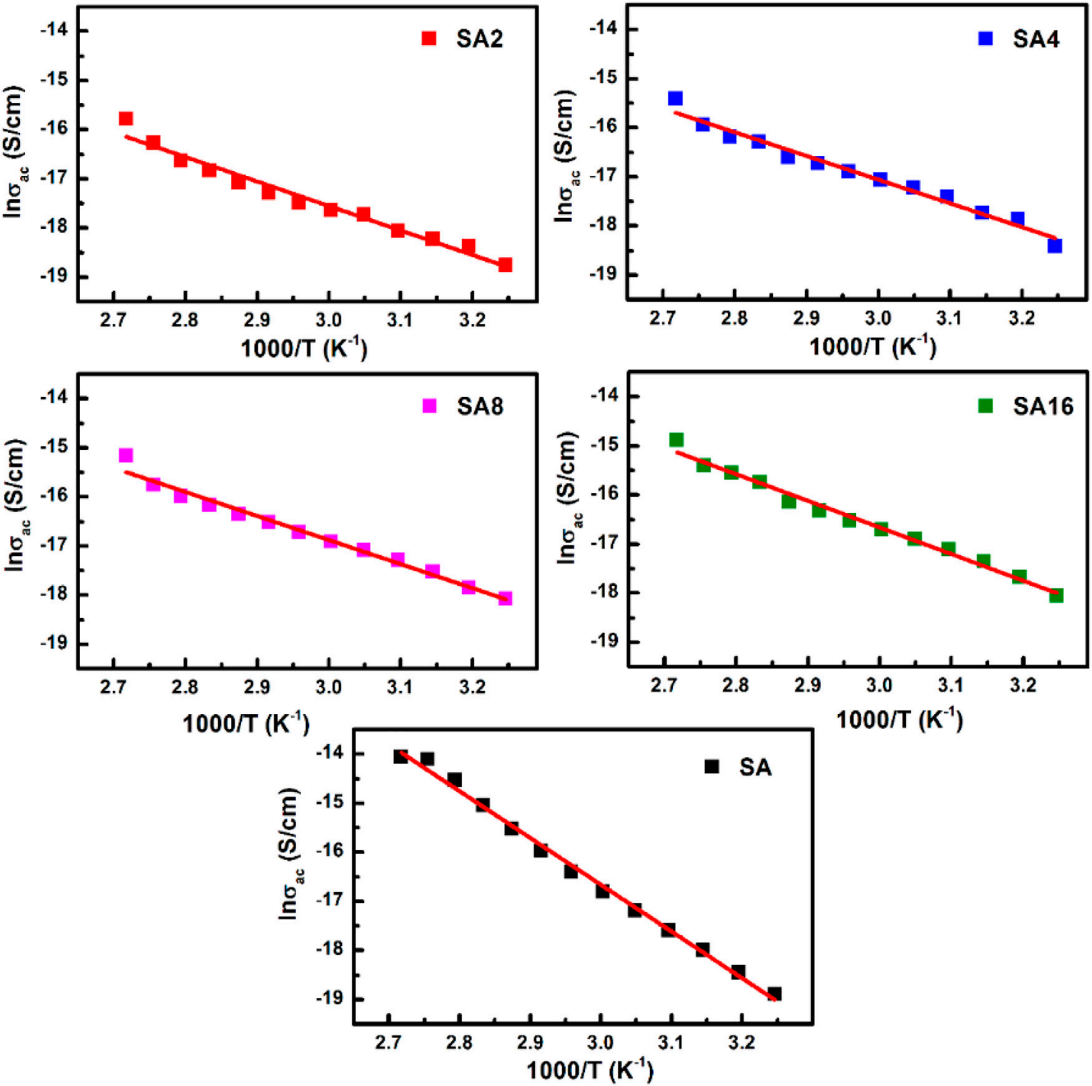
Arrhenius plots for different films.

Arrhenius plots for different films are shown in Figure 11. The slope of the plots is used to determine the activation energy (*E*_*a*_). Compared to that of the SA film, the *E*_*a*_ of the composite films is lower. This is due to the tunnelling phenomenon in the composite films (El-Ghamaz et al., 2016). The *E*_*a*_ of all the films tested is found to be less than 1eV. This order of magnitude is consistent with the observation made in other polymer composites in the literature. For example, the *E*_*a*_ of the composite consisting of LiClO_4_ NPs, sodium alginate and poly(vinyl alcohol) is in the range of 0.41–0.38 eV (Sheela et al., 2016); whereas that of the sodium lithium sulphide (NaLiS) composite generated from sodium alginate and NaLiS NPs is found to be around 0.31–0.75 eV (Shameem et al., 2018). The *E*_*a*_ *, ε′* and *σ*_*ac*_ of different films are summarized in Table 2.

**TABLE 2:** The *E*_*a*_, *ε’* and *σ*_*ac*_ of the film samples.

	SA	SA2	SA4	SA8	SA16
*ε′* at RT	34.04	68.64	82.09	85.27	88.34
*ε′* at 90°C	2,192	2,441	2,883	3,050	3,173
*σ*_*ac*_ at RT (10^−7^ S/cm)	14.941	7.224	6.637	15.542	8.881
*σ*_*ac*_ at 95°C (10^−7^ S/cm)	7.770	4.640	2.054	2.595	3.431
*E*_*a*_ (eV)	0.817	0.430	0.410	0.420	0.460

Abbreviation: RT, room temperature.

### Electrical Moduli of the Films

The reciprocal of complex permittivity is termed an electrical modulus. It is used to study space charge dielectric relaxation, the contribution of the electrode polarization effect, and the electrical conduction mechanism (Chandrakala et al., 2012; Chatterjee et al., 2015; Choudhary and Sengwa, 2017). The complex form of an electrical modulus is given by *M* ^*^ = *M'* + i*M"*, where *M′* is the real part and *M″* is the imaginary part of the modulus. *M′* and *M″* can be determined by using the following equations (Eq. 8 and Eq. 9):

$$M^{\prime }=\frac{\varepsilon^{\prime }}{\varepsilon^{\prime 2}\;+\;\varepsilon^{\prime \prime 2}}$$ (8)

$$M^{\prime \prime }=\frac{\varepsilon^{\prime \prime }}{\varepsilon^{\prime 2}\;+\;\varepsilon^{\prime \prime 2}}$$ (9)

**FIGURE 12:**
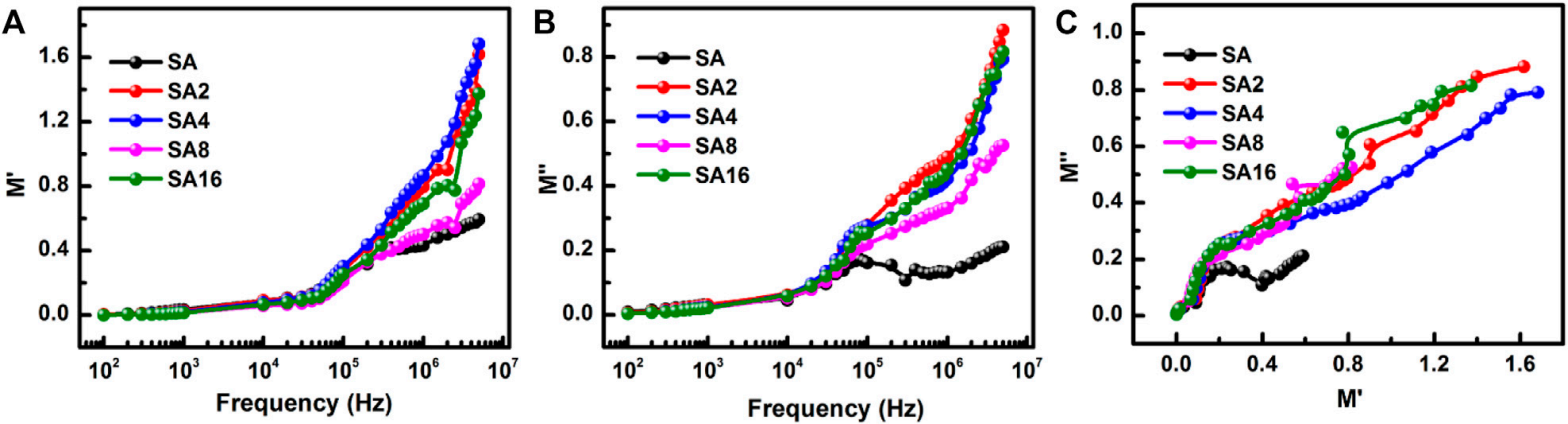
The frequency-dependence of **(A)** *M′* and **(B)** *M″* of different films at room temperature. **(C)** A plot of *M″* as a function of *M′* for different films at room temperature.

The frequency dependence of *M′* and *M″* of different films is shown in Figure 12. The value of *M′* is comparatively low at a lower frequency (Figure 12A). This is due to the suppression of interfacial polarization and to the short-range mobility of charge carriers (Chatterjee et al., 2015). When the frequency increases, *M′* increases. This indicates the presence of the electrode polarization effect at a higher frequency. In the case of *M″*, the magnitude shows an increasing trend with the frequency (Figure 12B), with the suppression of electrode polarization being one major mechanism explaining the low value of *M″* at a lower frequency (Choudhary and Sengwa, 2012; Sengwa and Choudhary, 2016). Furthermore, two different types of relaxation curves are found in our results. One is the complete relaxation curve shown by the SA film at 100 kHz. The other one is the partial relaxation curves shown by the composite films. The latter type of relaxation curves is related to interfacial polarization. In fact, electrical relaxation in polymer composites is affected by both the NPs and the polymer matrix. The phase transition of the polymer matrix (*α* relaxation) and the faster relaxation (*β*, *γ*) in the local segmental motion of the polymer chain contribute to the dielectric properties observed.

**FIGURE 13:**
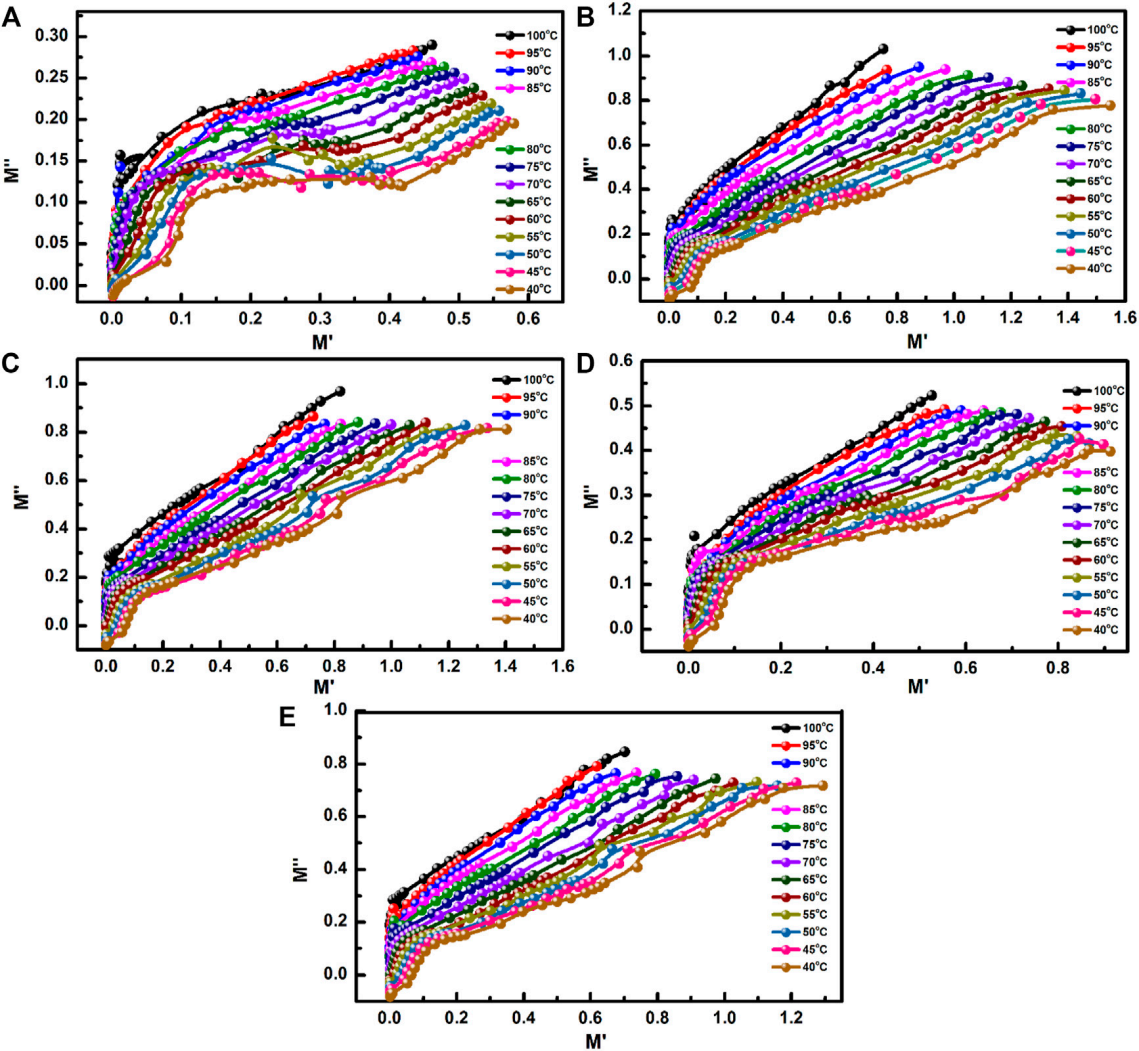
Plots of *M″* as a function of *M′* for the **(A)** SA film and the composite films [**(B)** SA2, **(C)** SA4, **(D)** SA8, and **(E)** SA16] at different temperatures.

The plot of *M″* as a function of *M′* is drawn to analyse the mechanism of electrical conductivity and dielectric relaxation. The plot is made in the frequency range of 100 Hz–5 MHz (Figure 12C). A complete relaxation peak is found in the plot for the SA film. For the composite films, the plots show partial relaxation peaks at lower *M′* and *M″* values and small peaks at higher *M′* and *M″* values. Because the centre of the imagined semi-circle in all the plots is below the *M′* axis, non-Debye relaxation occurs in all films tested. In the temperature range of 40–70°C, the plot for the SA film shows a complete relaxation peak; however, when the temperature increases beyond 70°C, partial relaxation is noted in the plot (Figure 13). Only partial relaxation is found in the plots for our composite films. Grain contribution is expected to play a more significant role than the contribution of grain boundaries during conduction (Yager, 1936; El-Sayed et al., 2013; Selmi et al., 2017). Because the centre of the relaxation peaks lies below the *M′* axis, non-Debye relaxation occurs in the composite films. LAO NPs in the composite films form a network with the polymer matrix through hydrogen bonding. The network increases the atomic packing density and hence the relaxation frequency (Chandrakala et al., 2012).

### Impedance Analysis of the Films

Impedance analysis can help understand the conduction behaviour of a composite and the interaction between the polymer and the particles incorporated inside. Impedance (Z*) is a complex number, with *Z′* and *Z″* being the real part and the imaginary part, respectively (Eq. 10).

$$Z^{*}=Z^{\prime }+iZ^{\prime \prime }$$ (10)

**FIGURE 14:**
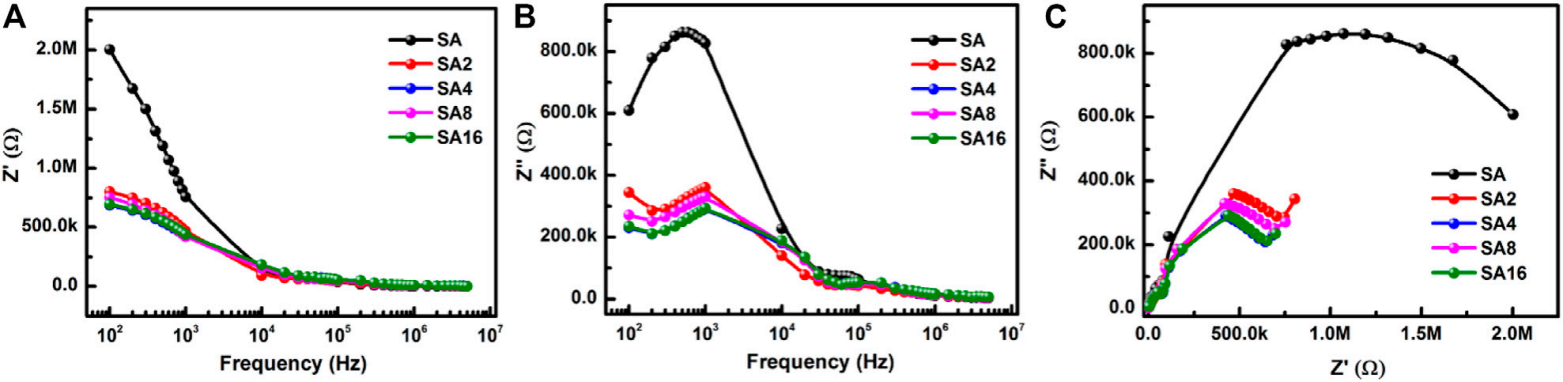
**(A)** A plot of *Z′* as a function of frequency for different films at room temperature. **(B)** A plot of *Z″* as a function of frequency for different films at room temperature. **(C)** An Nyquist plot for different films at room temperature.

The frequency dependence of *Z′* and *Z″* of different films at room temperature is shown in Figure 14. Our composite films show a decrease in *Z′* and *Z″* when the frequency increases. The high values of *Z′* and *Z″* at a lower frequency indicate the grain boundary contribution to the conduction process. When the frequency increases, the values of the impedance parameters decrease. This is due to a decline in space charge polarization, resulting in an increase in conductivity.

As far as the determination of the total electrical conduction behaviour exhibited by a material is concerned, both the grains and grain boundaries play a role. Information about the nature of the charge carriers is provided by a Nyquist plot, which is a plot of *Z″* as a function of *Z′* (Choudhary and Sengwa, 2015; Sheela et al., 2016; Correa et al., 2017). The Nyquist plot for different films at room temperature comprises two semi-circular arcs (Figure 14C). This indicates the semiconducting nature of the composite films. The semi-circle formed is equivalent to a parallel combination of bulk resistance (*R*_*b*_) and bulk capacitance (*C*_*b*_). *R*_*b*_ is contributed by the migration of charge carriers whereas *C*_*b*_ is caused by the immobility of the polymer chains. Based on the intersection point of a semi-circular arc and the *Z′* axis, *R*_*b*_ is determined from the Nyquist plot. Our results reveal that *R*_*b*_ decreases when the amount of LAO NPs incorporated into the film increases.

**FIGURE 15:**
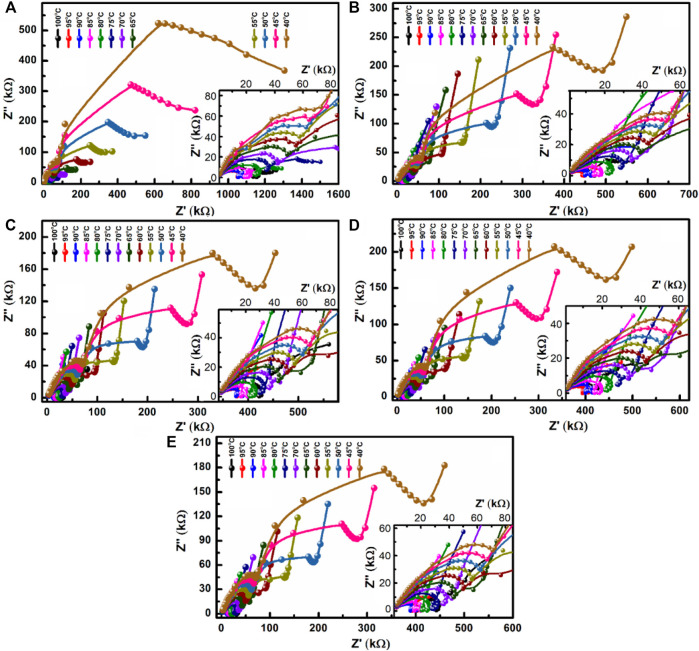
Nyquist plots for the **(A)** SA film and the composite films [**(B)** SA2, **(C)** SA4, **(D)** SA8, and **(E)** SA16] at different temperatures.

The Nyquist plots for different films at various temperatures are shown in Figure 15. In the plot for the SA film, a couple of semi-circular arcs are observed. The presence of these arcs is due to grain contribution at lower frequencies and grain boundary contribution at higher frequencies. Two well-defined regions (*viz.*, a semi-circular arc and an inclined peak) are found in the plots for the composite films. The high-frequency semi-circular arc represents the charge transfer process resulted from grain contribution; whereas the inclined peak represents the development of charges caused by polarization at the polymer interface. The broadness of the high-frequency semi-circular arc decreases when the temperature is elevated. This indicates the homogeneous nature of the films (Dias et al., 1996; Agrawal et al., 2019). The slope of the inclined peak is almost constant at different temperatures in all composite films tested, suggesting that no electrochemical reactions occur during conductivity measurements (Qian et al., 2002; Agrawal et al., 2019). In addition, *R*_*b*_ decreases with an increase in temperature in all films tested. Because the centre of the imagined semi-circular arc in the plots at different temperatures is below the *Z′* axis, this confirms the occurrence of non-Debye relaxation in our composite films.

## Conclusion

Polymer composites have been widely exploited over the years for use in different areas, ranging from the manufacturing of portable electronic devices to the fabrication of bioactive agent carriers. In this study, we have employed the method of ultrasound-assisted solution casting to generate composite films from SA and LAO NPs, with the NPs being fabricated by using the low-temperature hydrothermal method. Based on our results, various properties (including the degree of crystallinity and the thermal stability) of the SA film are enhanced upon the incorporation of the NPs. In addition, the presence of the NPs leads to an increase in the frequency-dependant *ε′* and *ε"* values at room temperature, and a decrease in *σ*_*ac*_. Not only do the electrical modulus spectra and Nyquist plots show that our composite films exhibit semiconducting nature and non-Debye relaxation, but they also reveal the effect of electrode polarization on electrical conduction in the films. All these demonstrate the good dielectric performance of our composite films. Along with the high biodegradability of SA as reported in the literature (Huq et al., 2012; Deepa et al., 2016; Salama et al., 2018), our films have high potential to become good candidates for possible microelectronic applications in the future.

## Data Availability

The original contributions presented in the study are included in the article/Supplementary Material, further inquiries can be directed to the corresponding author.
